# Solubility and Solvation Properties of Pharmaceutically Active Ionic Liquid Benzocainium Ibuprofenate in Natural Deep Eutectic Solvent Menthol–Lauric Acid

**DOI:** 10.3390/molecules28155723

**Published:** 2023-07-28

**Authors:** Jovana Panić, Maksim Rapaić, Slobodan Gadžurić, Milan Vraneš

**Affiliations:** Department of Chemistry, Biochemistry and Environmental Protection, Faculty of Sciences, University of Novi Sad, Trg Dositeja Obradovića 3, 21000 Novi Sad, Serbia; jovanap@dh.uns.ac.rs (J.P.); maxlab.ns@gmail.com (M.R.); milan.vranes@dh.uns.ac.rs (M.V.)

**Keywords:** ionic liquid, deep eutectic solvent, solubility, interactions

## Abstract

Due to their appealing physiochemical properties, particularly in the pharmaceutical industry, deep eutectic solvents (DESs) and ionic liquids (ILs) are utilized in various research fields and industries. The presented research analyzes the thermodynamic properties of a deep eutectic solvent created from natural molecules, menthol and lauric acid in a 2:1 molar ratio, and an ionic liquid based on two active pharmaceutical ingredients, benzocainium ibuprofenate. Initially, the low solubility of benzocainium ibuprofenate in water was observed, and a hydrophobic natural deep eutectic mixture of menthol:lauric acid in a 2:1 ratio was prepared to improve benzocainium ibuprofenate solubility. In order to determine the solvent properties of DESs and ILs mixtures at different temperatures and their molecular interactions to enhance the solvent performance, the apparent molar volume, limiting apparent molar expansibility, and viscosity B coefficient were estimated in temperature range from 293.15 K to 313.15 K and varying concentration of benzocainium ibuprofenate.

## 1. Introduction

Ionic liquids and deep eutectic solvents are types of solvents that have gained popularity in recent years due to their unique properties. Ionic liquids are the organic salts that are liquid below 100 °C, while deep eutectic solvents are a combination of two or more solid or liquid components linked by hydrogen bonds that form a eutectic mixture with a melting point lower than each component when mixed together [1]. These solvents have great potential in various areas such as green chemistry, energy storage, and pharmaceuticals due to their non-toxicity, non-flammability, high thermal stability, and great solvent power [1,2,3,4,5,6,7,8,9,10,11,12,13]. Today, pharmacy is a well-developed industry that deals with isolating or synthesizing various active components and producing medicaments based on them. The discovery and development of new drugs is an exponential process that goes through a difficult path where it encounters numerous problems. The most significant of them are poor solubility and stability of compounds, reduced bioavailability, lack of resources, and appropriate technologies, as well as polymorphism of active components [14,15,16]. Polymorphism represents the ability of a solid substance to exist in several different crystal forms. This is an extremely important characteristic of a compound because one form of a given compound can be extremely beneficial while another form can have an extremely negative or even toxic effect. It greatly compromises or impairs the effectiveness of a particular drug. One of the ways to overcome the aforementioned problems is the synthesis of pharmaceutically active compounds in the form of ionic liquids. The biggest advantage of ionic liquids and the reason for their use in the pharmaceutical industry is increased bioavailability or bioactivity, as well as designed lipophilicity that enables drug transport through the cell membrane [14,15,16,17]. It is precisely for these reasons that ionic liquids are increasingly the focus of research and application.

**Figure 1:**
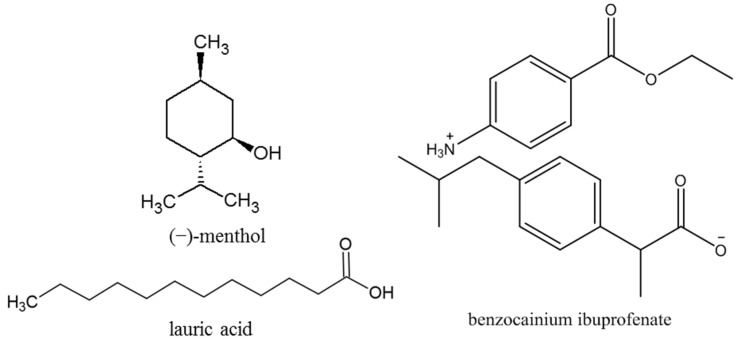
Chemical structures of the studied compounds.

In our previous study [18], ionic liquid benzocainium ibuprofenate with a melting temperature of 62 °C was synthesized and characterized (Figure 1). This compound represents the ionic liquid of the third generation since it completely consists of non-toxic and pharmacologically active ingredients. Benzocaine and ibuprofen are two common pain relievers that can be combined in a topical formula for effective pain relief. Benzocaine is a local anesthetic that works by numbing the area where it is applied, while ibuprofen is a non-steroidal anti-inflammatory drug (NSAID) that reduces inflammation and pain. Together, they could provide a dual-action approach to pain relief. After the synthesis of benzocainium ibuprofenate, its poor solubility in water was observed. Water is a common solvent used to dissolve drugs due to its ability to dissolve a wide range of molecules, including organic and inorganic compounds [19]. Many drugs are hydrophilic, meaning they dissolve readily in water and are therefore easily absorbed in the human body. Additionally, water is a safe and abundant solvent, making it an ideal choice for drug manufacturers. However, some drugs are not soluble in water, and alternative solvents are used in those cases. In recent years, numerous studies have explored the use of eutectic mixtures in pharmaceuticals to improve drug solubility and bioavailability [20,21]. In one study, the solubilities of nonsteroidal anti-inflammatory drugs such as aspirin, acetaminophen, ibuprofen, ketoprofen, and naproxen were investigated in the presence of deep eutectic solvents. The results showed that their solubility increased by 100 to 5400 times compared to an aqueous solution [22]. The emergence of natural deep eutectic solvents (NADES) as a new type of green solvent, following the advent of ionic liquids and deep eutectic solvents, brings many advantages such as sustainability, biocompatibility, environmental friendliness, and cheapness. Natural deep eutectic solvents possess extraordinary powers of dissolution and the ability to design desired properties, making them promising for applications in various fields. 

To find an appropriate solvent for benzocainium ibuprofenate, in our earlier publication, menthol and decanoic acid were mixed in different molar ratios forming natural deep eutectic solvents [18]. As a continuation, in this work, the natural deep eutectic mixture menthol:lauric acid in a 2:1 molar ratio will be examined as a potential solvent for the investigated ionic liquid (Figure 1). A molar ratio of 2:1 for menthol and lauric acid in a prepared mixture was chosen since Ribeiro et al. reported that menthol and lauric acid form deep eutectic solvent at that molar ratio [23]. In a natural deep eutectic solvent, lauric acid functions as a hydrogen bond donor, while menthol serves as a hydrogen acceptor. The solvent’s physical properties, including its low viscosity and high potential for enhancing the solubility of hydrophobic active pharmaceutical ingredients, make it an attractive alternative to traditional solvents. Lauric acid, as a fatty acid, has been known to possess antimicrobial properties and has been used in various applications such as a natural preservative and skincare products [24], while menthol is used for pain relief and minor muscle and joint pain [25]. Lauric acid and menthol are inexpensive compounds with a long shelf life; they are non-toxic and safe to handle. This combination is commonly used to soothe sore muscles and joint pain and alleviate symptoms associated with minor skin irritations such as insect bites or rashes. It can be found in various topical products such as creams, lotions, and balms, and is considered safe for most people when used as directed. Both menthol and lauric acid are derived from natural sources, making this solvent environmentally friendly. The combination of ionic liquids based on known drugs and a natural deep eutectic solvent designs a versatile pharmacological system that could serve as an affordable solution for local or transdermal application. Topical drug delivery systems have numerous advantages when compared with other delivery systems (oral and parenteral), namely, the avoidance of significant systemic metabolism, thus lowering the required daily doses, providing better patient compliance and even economic benefits. Application of local anesthetics can provoke skin irritation or allergic reactions to topical anesthetics, causing redness, itching, rash, or swelling at the application site. However, applying menthol-lauric acid DES can help to overcome these issues. Menthol has anti-itching properties, activating cold receptors in the skin and creating a cooling sensation upon application. This can provide temporary relief from itching caused by allergic reactions or skin irritations. Additionally, lauric acid’s moisturizing and emollient effects make applying the local anesthetics more comfortable. While topical local anesthetics are intended to provide anesthesia only to the skin’s surface layers, they can be absorbed through the skin and enter the bloodstream, especially if applied to a large area. This can potentially lead to systemic side effects such as dizziness, drowsiness, headache, or even systemic toxicity. Our previous research has shown that using excess menthol with decanoic acid (DES with a 2:1 molar ratio of menthol:decanoic acid) prevents significant solvation properties of this DES and enables significant interactions between cations and anions of the benzocainium ibuprofenate ionic liquid dissolved in it. This results in the ionic liquid mainly being in the form of ion pairs with a high lipophilic character. The absence of dissociation of the ionic liquid in the examined DES can prevent it from entering the bloodstream.

Further research is being conducted to explore the full potential of this natural deep eutectic solvent. The aim of the research was to prepare a natural deep eutectic solvent menthol:lauric acid in a 2:1 molar ratio and then determine the solubility and explain interactions of benzocainium ibuprofenate in the prepared mixture. Additionally, the solubility of benzocainium ibuprofenate in pure water was measured and compared with obtained results. The densities and viscosities of the investigated ionic liquid in natural deep eutectic solvent menthol:lauric acid in a 2:1 molar ratio as a solvent were measured in the temperature range from 293.15 to 313.15 K at different ionic liquid molalities. The findings from this work will shed light on the interaction between the ionic liquid benzocainium ibuprofenate and the menthol:lauric acid mixtures, the impact of temperature on viscosity and density, the ordering of the solvent and solute molecules, and the structure-making/breaking properties of the ionic liquid. 

## 2. Results and Discussion

### 2.1. Characterization of Prepared Solvent

**Figure 2:**
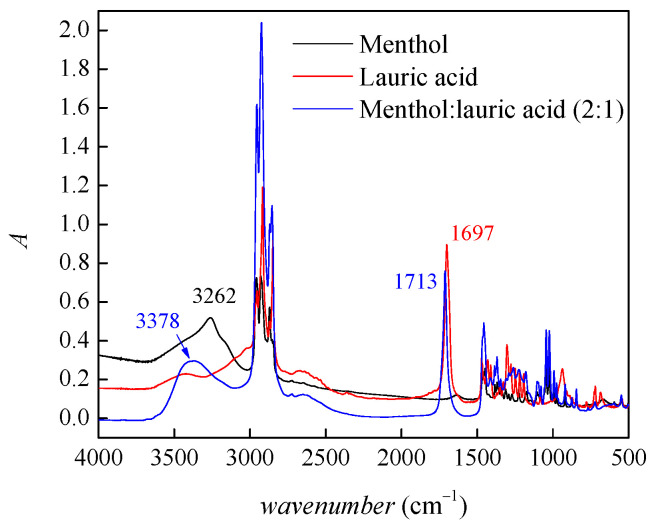
Infrared spectra of starting compounds and menthol:lauric acid mixture in molar ratio 2:1.

The research focused on verifying whether a natural deep eutectic solvent made from menthol and lauric acid is a suitable solvent for the ionic liquid benzocainium ibuprofenate. After solvent preparation, the structure of the prepared natural deep eutectic solvent was confirmed by infrared (IR) spectroscopy. The recorded IR spectra of starting compounds, menthol and lauric acid, and a prepared menthol:lauric acid mixture in a molar ratio of 2:1 are shown in Figure 2. From Figure 2, peaks in the range of 3300–2500 cm^−1^ representing stretching vibrations of the O-H group can be observed. It can be seen that the peak of the menthol O-H group is shifted from 3262 cm^−1^ to a higher wavenumber of 3378 cm^−1^ in the mixture menthol:lauric acid 2:1, and that its intensity decreased, which indicates the formation of a hydrogen bond. Moreover, a shift and decrease in the intensity of the peak originating from the stretching vibration of the carbonyl group of lauric acid from 1697 cm^−1^ to 1713 cm^−1^ in the mixture menthol:lauric acid 2:1 is observed, which also indicates the formation of a hydrogen bond. Using Carl Fisher coulometric titration, it was determined that the prepared natural deep eutectic solvent contains about 9 ppm of water. 

### 2.2. Solubility Determination

To assess the efficiency of solubility of benzocainium ibuprofenate in water and deep eutectic solvent menthol:lauric acid 2:1 at the temperature of 293.15 K, the saturation shake flask technique was applied [26]. Solubility results represent the amount of benzocainium ibuprofenate in solution in equilibrium with a solid, determined by measuring the concentration of benzocainium ibuprofenate in saturated solution using the high-performance liquid chromatography with diode-array detection (HPLC-DAD) technique. The obtained solubility values of 0.0006 mol·dm^−3^ in water and 0.4182 mol·dm^−3^ in menthol:lauric acid 2:1 mixture suggest that the chosen natural deep eutectic mixture is a viable solvent for investigating the interactions between benzocainium ibuprofenate and mixture molecules.

### 2.3. Volumetric and Viscosity Studies

**Figure 3:**
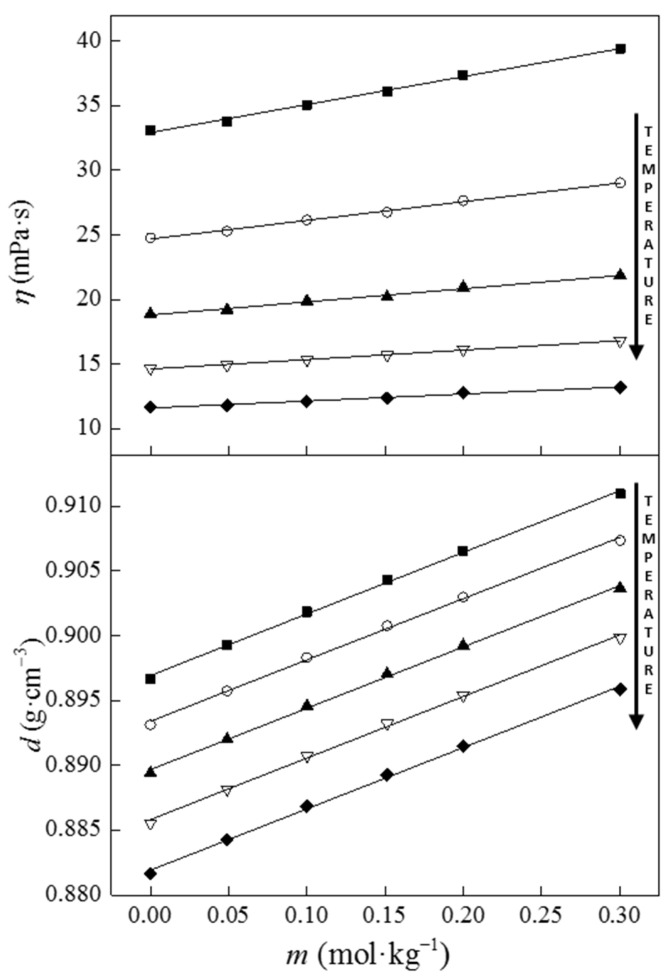
The change of density and viscosity values for the solution of benzocainium ibuprofenate in deep eutectic solvent menthol:lauric acid in a 2:1 molar ratio with different molalities of benzocainium ibuprofenate at different temperatures: *T* = (■), 293.15; (○), 298.15; (▲), 303.15; (▽), 308.15; and (◆) 313.15 K.

To examine the interactions between pharmaceutically active benzocainium ibuprofenate and natural deep eutectic mixture with menthol and lauric acid in a molar ratio of 2:1, volumetric and viscosimetric measurements were conducted. The obtained values are given in Tables S1 and S2 in the Supplementary Materials and graphically presented in Figure 3. The densities and viscosities of the prepared solutions at various temperatures and different molality levels of the ionic liquid were tested (Figure 3). An increase in the density and viscosity values has been observed with enhancements to the concentration of benzocainium ibuprofenate, while increasing the temperature has the opposite effect. 

From the experimental densities, the apparent molar volume (*V*_ϕ_) of the solutes was calculated (Table S3) using the Equation (S1) as provided in the Supplementary Materials. Obtained values were fitted with Masson’s Equation (1) [27]:

*V*_ϕ_ = *V*_ϕ_^o^ + *S*_v_*c*^1/2^. (1)

**Figure 4:**
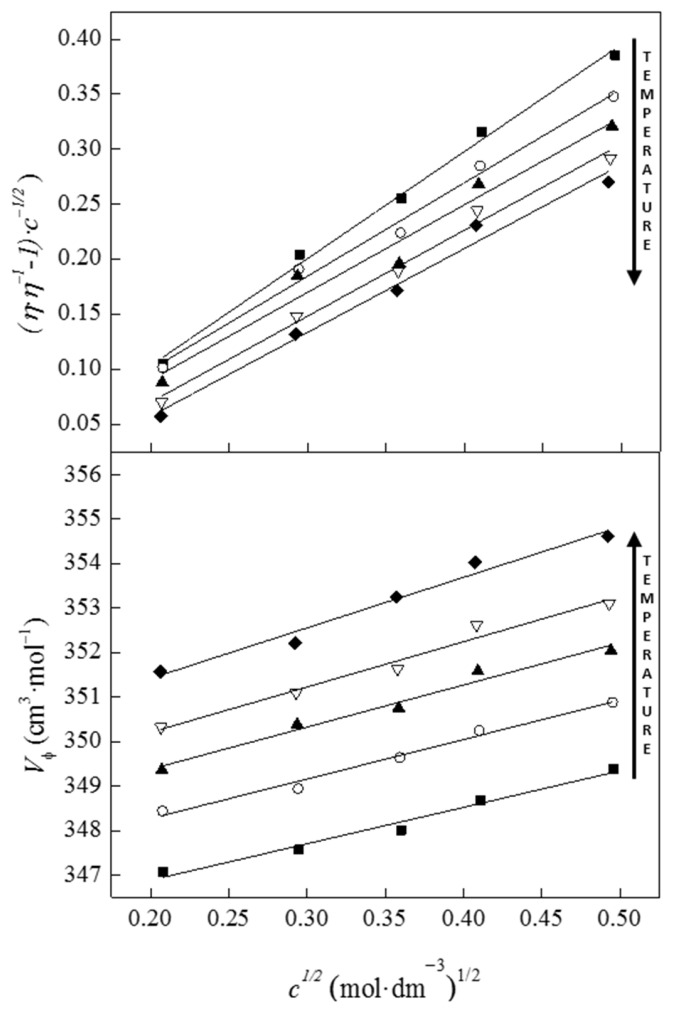
Plots of the apparent molar volume (*V*_ϕ_) and relative viscosity (*η* / *η*_o_ – 1) – *c*^1/2^ as a function of square root of concentration at different temperatures: *T* = (■), 293.15; (○), 298.15; (▲), 303.15; (▽), 308.15; and (◆) 313.15 K.

The plot of the apparent molar volume (*V*_ϕ_) versus square root of concentration (*c*^1/2^) was linear, as illustrated in Figure 4. In Masson’s equation, *V*_ϕ_^o^ represents the apparent molar volume of the solute at infinite dilution, while *S*_v_ is the experimental slope, which measures the strength of solute–solute interactions. The apparent molar volume at infinite dilution, *V*_ϕ_^o^, shows how much the volume of the solution changes after adding 1 mole of solute to an infinitely large volume of solvent. Values of *V*_ϕ_^o^ are obtained by extrapolating the corresponding apparent molar volumes to infinite dilution, where the interactions between the solutes are negligible. It takes into account the changes in volume that occur upon dissolution, such as the breaking of intermolecular bonds and the formation of new solute–solvent interactions. The calculated values of *V*_ϕ_^o^ and *S*_V_ are listed in Table 1.

As seen in Figure 4, the apparent molar volume values of benzocainium ibuprofenate in menthol:lauric acid 2:1 mixture increase as the solute concentration increases, regardless of the applied temperature. In order to compare the effect of extending the alkyl chain in carboxylic acid structure on NADES’ solvation ability, the results presented in this study will be compared with those obtained in our recent work [18]. In our recent work [18], a mixture of menthol and decanoic acid was used to dissolve benzocainium ibuprofenate in the same molar ratio as the menthol:lauric acid used in this study. Calculated values of the apparent molar volume of the solute at infinite dilution (*V*_ϕ_^o^) in menthol:lauric acid are lower compared to those observed in menthol:decanoic acid 2:1 as a natural, deep eutectic solvent at the same temperature [18]. These results indicate that adding one mole of ionic liquid to an infinitely large volume of menthol:lauric acid has less effect on volume expansion than natural deep eutectic solvent made from menthol:decanoic acid. The lower values of the apparent molar volume of the solute at infinite dilution in the lauric acid system may be due wither to stronger interactions between the ionic liquid and the solvent components or to their better steric fitting. 

Positive *S*_v_ values are common in solutions with strong interactions between solute molecules, whereas negative *S*_v_ values indicate weak interactions between solutes. Thus, based on the obtained *S*_v_ values in Table 1, interactions between benzocainium cations and ibuprofenate anions are significant. In the menthol:decanoic acid 2:1 system, *S*_v_ values are lower at all temperatures (range of *S*_V_ values from 2.10 to 1.86 cm^3^·kg^−1^·mol^−2^ in the same temperature range) [18]. It can be concluded that the extension of the alkyl chain for the ethylene group in the fatty acid as a solvent component leads to stronger interactions between the ions of the ionic liquid solute.

**Figure 5:**
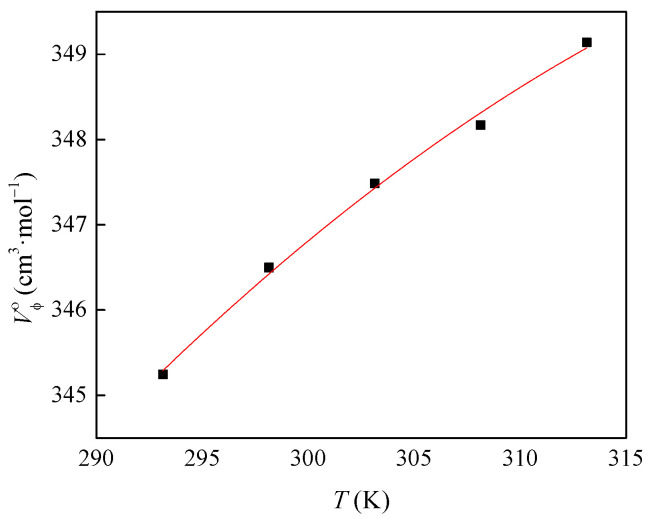
Variation of apparent molar volume at infinite dilution (*V*_ϕ_^o^) of benzocainium ibuprofenate in deep eutectic solvent menthol:lauric acid in molar ratio of 2:1 with a temperature (*V*_ϕ_^o^ = *a*_o_ + *a*_1_*T* + *a*_2_*T*^2^).

The values of *V*_ϕ_^o^ were fitted with a second-order equation (*V*_ϕ_^o^ =*a*_o_ + *a*_1_*T* + *a*_2_*T*^2^) depending on the temperature (Figure 5) in order to calculate limiting apparent molar expansibilities (*E*_φ_^o^) using the obtained coefficients (Equation (2)).

(2) $$E_{\phi }^{o}={\left(\frac{\partial V_{\phi }^{o}}{\partial T}\right)}_{p}=a_{1}+2a_{2}T$$

The obtained values of fitting coefficients *a*_o_, *a*_1_, and *a*_2_ amount 61.703 cm^3^·mol^−1^, 1.700 cm^3^·mol^−1^·K^−1^, and −0.002 cm^3^·mol^−1^·K^−2^, respectively. The calculated limiting apparent molar expansibilities are presented in Table 1. The values of the limiting apparent molar expansibilities are positive, which indicates that the solvent molecules move away from the ionic liquid more quickly than from each other. With increasing temperature, the release of solvent molecules from the solvation sphere of the ionic liquid is less pronounced.

The solution viscosities were analyzed using the Jones–Dole Equation (3) [28] (presented in Figure 4), and fitting parameters are given in Table 1.

(3) $$\frac{(\eta /\eta_{o})-1}{c^{1/2}}=A+Bc^{1/2}$$

In this equation, *η*_o_ is the viscosity of the pure menthol:lauric acid mixture, coefficient *A* is related to electrostatic interactions, and coefficient *B* is a useful measure of ion–solvent interactions and is designated as one of the prevalent criteria for the determination of solvent organization around solute. The positive values of coefficient *B*, which decrease with increase in the temperature, indicate the structure-making ability of benzocainium ibuprofenate in menthol:lauric acid mixture. By comparing the *B* coefficient of the ionic liquid in the menthol–decanoic with menthol–lauric acid natural deep eutectic solvent [18], it was concluded that the presence of the ionic liquid significantly increases the structural organization of the solvent with decanoic acid at lower temperatures. With an increase in temperature, the structure-making properties of ionic liquids are more pronounced in the system with lauric acid. A comparison of the viscosity *B* coefficients of lauric and decanoic acid [18] at different temperatures is presented in Figure S1.

## 3. Materials and Methods

### 3.1. Solvent Preparation

Natural deep eutectic solvent menthol:lauric acid was prepared by weighing (Precisa 92SM-202A, Dietikon, Switzerland, Δ*m* = ±1 × 10^−5^ g) and mixing suitable amounts (-)-menthol (CAS number: 2216-51-5, mass fraction purity ≥0.99) and lauric acid (CAS number: 143-07-7, mass fraction purity ≥0.99) in a molar ratio of 2:1 under stirring at 60 °C for 24 h at ambient conditions, in which a single liquid phase was formed and maintained then cooled to room temperature. Prepared samples were dried under vacuum in a rotary evaporator and stored in vacuum driers. The residual water content in the prepared mixture is determined in triplicate using an 831 coulometric Karl Fischer titrator (Metrohm, Herisau, Switzerland). After preparation of natural deep eutectic mixtures, infrared spectroscopy was used to check the eutectic mixtures structures and to confirm the interaction between two compounds leading to the eutectic formation.

### 3.2. Spectroscopy Analysis

Infrared spectra were recorded as neat samples from (4000 to 650) cm^−1^ on a Thermo Nicolet Nexus 670 spectrometer (Thermo Fisher Scientific Inc., Waltham, MA, USA) fitted with a Universal ATR Sampling Accessory (Thermo Fisher Scientific Inc., Waltham, MA, USA). The measurements were performed with a total of 60 scans, at *T* = 298.15 K, and with a spectral resolution of 2 cm^−1^. The software package Omnic version 6.2 was used in the data acquisition and spectral analysis. 

### 3.3. Solubility Determination

**Figure 6:**
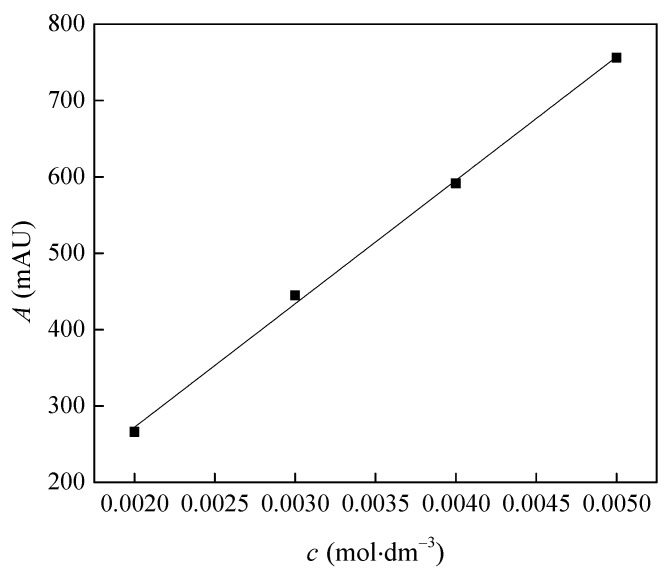
Calibration curve for benzocainium ibuprofenate solubility determination.

The equilibrium/saturated solubility of benzocainium ibuprofenate (synthesized and characterized in the previous paper [18]) in water and menthol:lauric acid was determined at 298.15 K via the reported “saturation shake flask technique” described elsewhere [26]. To achieve solution supersaturation, an excessive amount of each ionic liquid (g) was added to 5 mL of ultrapure deionized water in 10 mL glass vials and in 10 mL of natural deep eutectic solvent menthol:lauric acid in molar ratio 2:1. The experiment was repeated in triplicate (*n* = 3.0). The saturated solution was then placed in a shaker and stirred at 200 rpm for 24 h at a controlled temperature of 298.15 K (±0.1 K). After stirring, the samples were allowed to settle and separate into two phases for the next 24 h at the same temperature. Once the two phases had completely separated, the upper phase from each vial was carefully taken, filtered through a 0.25 μm syringe filter to remove potential impurities, diluted in mobile phase, and assayed by HPLC. An HPLC method was used to determine the concentration of ibuprofen in the saturated solution using a reversed-phase Agilent 1290 system (Agilent Technologies, Santa Clara, CA, USA) with a diode array detector (DAD) and a Poroshell 120 EC-C18 (4.6 × 50 mm 2.7 µm) column (Agilent Technologies, Santa Clara, CA, USA). The detection of ibuprofen was at 230 nm. The solubility was calculated based on the calibration curve prepared for ibuprofen (CAS number: 15687-27-1, mass fraction purity ≥0.98) (Figure 6). Mobile phase A was 0.3% phosphoric acid; and mobile phase B was acetonitrile. Isocratic elution was applied with 30% mobile phase A and 70% mobile phase B over 3.0 min at the flow rate of 2.0 cm^3^/min. The column temperature was 40 °C, and the injection volume was 20 μL.

### 3.4. Density and Viscosity Measurements

**Figure 7:**
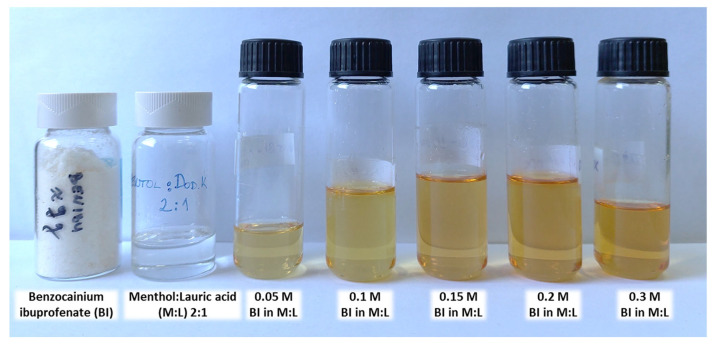
The appearance of the prepared ionic liquid, natural deep eutectic solvent, and prepared IL-NADES mixtures.

Mixtures of benzocainium ibuprofenate and menthol:lauric acid as a solvent were prepared in various concentrations (Figure 7). 

The prepared solvent and the mixed systems of benzocainium ibuprofenate and menthol:lauric acid were analyzed by using the vibrating tube Rudolph Research Analytical DDM 2911 densimeter (Hackettstown, NJ, USA) and Brookfield Viscometer DV II + Pro (Middleborough, MA, USA) with the spindle type SC4-18 and the rotation speed from 20 to 70 RPM. The instruments were thermostated within ±0.01 K. Before each series of measures, calibration of the densimeter was performed at the atmospheric pressure (*p* = 0.1 Mpa) using ambient air and bi-distilled ultrapure water in the temperature range from 293.15 to 353.15 K. A viscometer cell was protected from moisture with the manufacturer’s compartment and calibrated using the liquids of different viscosities purchased from the manufacturer. The estimated uncertainty in the density measured was 3.0 × 10^−3^ g∙cm^−3^, while the viscosity relative standard uncertainty was about 1.5%. The density and viscosity readings were obtained for each sample for temperatures ranging from 293.15 K to 313.15 K. Presented experimental values are the mean of three measurements. 

## 4. Conclusions

The formation of hydrogen bonds in prepared natural deep eutectic solvent menthol:lauric acid in molar ratio 2:1 was confirmed by Infrared spectroscopy. The solubility of the ionic liquid benzocainium ibuprofenate is about 700 times higher in the natural deep eutectic solvent menthol:lauric acid 2:1 compared to pure water at 293.15 K. The obtained positive *S*_v_ values point out significant interactions between benzocainium cations and ibuprofenate anions. The positive values of the limiting apparent molar expansibilities indicate that the solvent molecules move away from the ionic liquid more quickly than from each other. Further, as the temperature rises, the ionic liquid has a weaker tendency to release solvent molecules from its solvation sphere. Positive values of the viscosity coefficient *B* and negative temperature coefficient indicate the structure-making properties of benzocainium ibuprofenate in a prepared natural deep eutectic solvent. Based on the obtained values of viscosity and density of the benzocainium ibuprofenate in menthol:lauric acid mixtures, it can be concluded that there are strong interactions between the ions of the ionic liquid in the prepared mixtures. Comparing the results with those obtained in the menthol:decanoic acid system 2:1, it can be concluded that the variation in the length of the fatty acid chain, as a component of natural deep eutectic solvent, leads to a change in the strength of interactions between ions from the ionic liquid. The ability to fine-tune the strength of the interactions between the pharmaceutically active ions of the ionic liquid by selecting the appropriate fatty acid in NADES as a solvent is a promising strategy for developing multicomponent pharmaceutical products for topical application. 

## Figures and Tables

**Table 1:** Calculated thermodynamic parameters: apparent molar volume of the solute at infinite dilution (*V*_ϕ_^o^), *S*_V_ coefficient, limiting apparent molar expansibility (*E*_φ_^o^), and *A* and *B* viscosity coefficient for the solution of benzocainium ibuprofenate in deep eutectic solvent menthol:lauric acid in a 2:1 molar ratio.

*T* (K)	293.15	298.15	303.15	308.15	313.15
*V*_ϕ_^o^ (cm^3^·mol^−1^)	345.24	346.50	347.49	348.17	349.14
*S*_V_ (cm^3^·dm^3/2^·mol^−3/2^)	8.20	8.87	9.46	10.19	11.37
*E*_φ_^o^ (cm^3^·mol^−1^·K^−1^)	0.2390	0.2142	0.1893	0.1645	0.1396
*A* (dm^3/2^·mol^−1/2^)	−0.0913	−0.0703	−0.0690	−0.0865	−0.0951
*B* (dm^3^∙mol^−1^)	0.9727	0.8494	0.7960	0.7820	0.7619

## Data Availability

Data are available from the authors if required.

## Supplementary Materials

The following supporting information can be downloaded at: https://www.mdpi.com/article/10.3390/molecules28155723/s1: Table S1: Experimental values of density (*d*) of benzocainium ibuprofenate in deep eutectic solvent menthol:lauric acid in molar ratio of 2:1 at different temperatures and IL molalities; Table S2: Experimental values of viscosity (*η*) of benzocainium ibuprofenate in deep eutectic solvent menthol:lauric acid in molar ratio of 2:1 at different temperatures and IL molalities; Table S3: Calculated values of apparent molar volumes (*V*_ϕ_) of benzocainium ibuprofenate in deep eutectic solvent menthol:lauric acid in molar ratio of 2:1 at different temperatures and IL molalities. Figure S1: Comparison of viscosity B coefficients of lauric and decanoic acid [8] at different temperatures.
