# Supplementary material for: Solubility and Solvation Properties of Pharmaceutically Active Ionic Liquid Benzocainium Ibuprofenate in Natural Deep Eutectic Solvent Menthol–Lauric Acid
Source: Molecules. 2023 Jul 28;28(15):5723. doi: 10.3390/molecules28155723 (PMC10420925; doi:10.3390/molecules28155723)
Supplement: Supplementary file 1 [file molecules-28-05723-s001.zip › molecules-2511389-supplementary.pdf]

Supporting Materials

Solubility and Solvation Properties of Pharmaceutically Active Ionic Liquid Benzocainium Ibuprofenate in Natural Deep Eutectic Solvent Menthol–Lauric Acid

Jovana Panić, Maksim Rapaić, Slobodan Gadžurić * and Milan Vraneš

Department of Chemistry, Biochemistry and Environmental Protection, Faculty of Sciences, University of Novi Sad, Trg Dositeja Obradovića 3, 21000 Novi Sad, Serbia; jovanap@dh.uns.ac.rs (J.P.); maxlab.ns@gmail.com (M.R.); milan.vranes@dh.uns.ac.rs (M.V.)

* Correspondence: slobodan.gadzuric@dh.uns.ac.rs

Table S1. Experimental values of density (d) of benzocainium ibuprofenate in deep eutectic solvent menthol:lauric acid in molar ratio of 2:1 at different temperatures and IL molalities.

m (mol·kg^{-1})	T (K)				
	293.15	298.15	303.15	308.15	313.15
	d (g·cm^{-3})				
0.0000	0.89665	0.89313	0.88940	0.88554	0.88163
0.0490	0.89926	0.89573	0.89201	0.88816	0.88425
0.1000	0.90185	0.89831	0.89456	0.89074	0.88684
0.1513	0.90435	0.90077	0.89705	0.89323	0.88928
0.2000	0.90658	0.90299	0.89924	0.89541	0.89148
0.3003	0.91097	0.90737	0.90365	0.89983	0.89587

Table S2. Experimental values of viscosity (η) of benzocainium ibuprofenate in deep eutectic solvent menthol:lauric acid in molar ratio of 2:1 at different temperatures and IL molalities.

m (mol·kg^{-1})	T (K)				
	293.15	298.15	303.15	308.15	313.15
	η (mPa·s)				
0.0000	33.08	24.77	18.87	14.70	11.67
0.0490	33.80	25.29	19.21	14.91	11.81
0.1000	35.07	26.16	19.89	15.33	12.12
0.1513	36.12	26.76	20.19	15.69	12.39
0.2000	37.37	27.66	20.94	16.16	12.77
0.3003	39.41	29.03	21.86	16.81	13.22

Table S3. Calculated values of apparent molar volumes (V_Φ) of benzocainium ibuprofenate in deep eutectic solvent menthol:lauric acid in molar ratio of 2:1 at different temperatures and IL molalities.

m (mol·kg^{-1})	T (K)				
	293.15	298.15	303.15	308.15	313.15
	V_Φ (cm^3·mol^{-1})				
0.0490	347.08	348.44	349.36	350.32	351.57
0.1000	347.57	348.94	350.38	351.09	352.21
0.1513	348.01	349.64	350.75	351.63	353.25
0.2000	348.67	350.25	351.58	352.62	354.03
0.3003	349.39	350.88	352.04	353.10	354.61

From the experimental densities the apparent molar volumes, V_ϕ, were calculated using the equation:

$$V_\phi = \frac{1000(d_2 - d)}{mdd_2} + \frac{M_1}{d} \quad \text{(S1)},$$

Where m (mol·kg^{-1}) is molality of solute, d (g·cm^{-3}) the experimental density of the solution, while d_2 is related to the solvent,and M_1 (g·mol^{-1}) is a molar mass of solute.

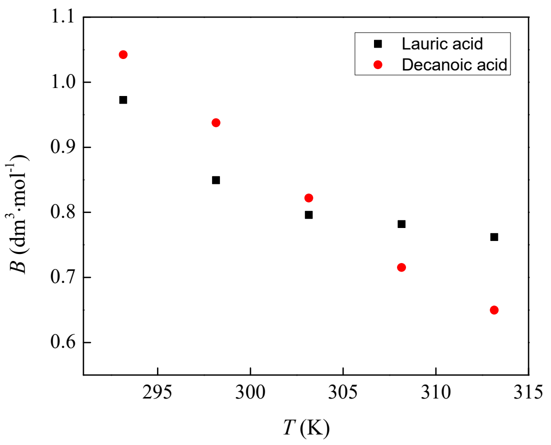

Figure S1. Comparison of viscosity *B* coefficients of lauric and decanoic acid [8] at different temperatures.
